# A bacterial pangenome association study identifies a role for polyamine metabolism and alkaline tolerance in middle ear infections

**DOI:** 10.1371/journal.ppat.1014649

**Published:** 2026-09-25

**Authors:** Jocelyn A. Hammond, Rachel L. Ehrlich, Ariel Gonzalez, Begoña Euba, Kevin M. Raible, Jill M. Lawrence, Ari B. Gordon, Evangeline Williams, Mary C. Marino, Jason Limbo, Steven Lang, Bhaswati Sen, Sergey V. Balashov, Azad Ahmed, Junkal Garmendia, Joris Beld, Garth D. Ehrlich, Michael E. Pichichero, Ravinder Kaur, Joshua Chang Mell

**Affiliations:** 1 Department of Microbiology & Immunology, Drexel University College of Medicine; Center for Genomic Sciences, Institute of Molecular Medicine and Infectious Disease; Drexel University, Philadelphia, Pennsylvania, United States of America; 2 Center for Infectious Diseases and Immunology, Rochester General Hospital Research Institute, Rochester General Hospital, Rochester, NewYork, United States of America; 3 Instituto de Agrobiotecnología, Consejo Superior de Investigaciones Científicas-Gobierno de Navarra, Mutilva, Spain; 4 Centro de Investigación Biomédica en Red Enfermedades Respiratorias (CIBERES), Madrid, Spain; 5 Conexión Antimicrobial Resistance-AMR, CSIC, Madrid, Spain; 6 Department of Otolaryngology – Head and Neck Surgery; Drexel University College of Medicine, Philadelphia, Pennsylvania, United States of America; University of Melbourne, AUSTRALIA

## Abstract

Infection of the middle ear, or otitis media (OM), is a disease that commonly afflicts children, and *Haemophilus influenzae* is the most prevalent OM-associated bacterium. We sought new insights into mechanisms of pathogenesis by identifying bacterial genes associated with OM disease. We whole-genome sequenced >200 clinical isolates collected over time from 9 healthy and 12 OM-prone children. Genome analysis first annotated and clustered homologous genes from across assemblies, inferred the phylogeny, and identified closely related set of strains, or clonal lineages, finding both transient and persistent colonization by distinct lineages. Phylogeny-informed association tests then identified 7 strong associations of genes/operons with health or disease. Remarkably, 5 associations were with gene absences and OM, and all 5 had predicted functions consistent with roles in pH homeostasis and nitrogen metabolism, specifically in transport and modification of polyamines. Using a laboratory strain, knockouts of candidate “anti-OM” operon *potE-speF* were tested. *In vitro* growth assays varying pH and supplementation with polyamines showed that the presence of *potE-speF* conferred alkaline sensitivity to cells in the presence of ornithine. Measurements of human middle ear fluid pH found higher alkalinity in specimens where *H. influenzae* was collected over those with other OM pathogens, supporting the hypothesis that the alkaline middle ear microenvironment in OM arises from or selects for *H. influenzae* infection. Experiments using a mouse OM model found that absence of *potE-speF* caused a ~ 63-fold increase in bacterial disease burden in middle ear washes. By contrast, a mouse lung infection model showed no significant difference. We conclude that *H. influenzae* lacking *potE-speF* have a selective advantage in OM disease pathogenesis, because these strains thrive in the otherwise hostile alkaline environment that occurs in the infected middle ears of children.

## Author summary

Many bacteria that inhabit the healthy human microbiome also cause disease. How bacteria transition into pathogens remains poorly understood and results from a complex interplay among microbial, host, and environmental factors. Among such bacteria are species that are found in the healthy human nasopharynx but also cause middle-ear infections (otitis media, or OM), as well as other diseases. Most people experience at least one OM episode in early childhood, and many have persistent disease with potentially lifelong consequences from delayed language acquisition or hearing loss. Here we present evidence that the absence of certain genes of the bacterium *Haemophilus influenzae* is associated with OM disease. Prior work showed that children with recurrent OM who later receive tympanostomy tube surgery often have a middle ear microenvironment characterized by alkaline pH. Here we show that the absence of a two-gene unit involved in polyamine transport and conversion, *potE-speF*, results in an alkaline-tolerant phenotype. We show that strains without these genes increase the bacterial disease burden 63-fold in a mouse model of OM but not in a lung infection model. The results point to unexplored host-mediated mechanisms for limiting pathogen burden by creating an alkaline environment in the middle ears of children.

## Introduction

Many host-associated bacteria normally behave as commensal colonizers but also cause high morbidity due to their role in pathogenic infections. The risk that such microbes transition to pathogens depends on a complex interplay of host, environmental, and bacterial influences. These include delayed or compromised immune functions, predisposing chronic conditions, co-infections by viruses or other pathogens, and the extensive genetic and phenotypic variation among bacterial strains [1–5]. Identifying the naturally occurring bacterial genetic variants that influence disease risk will provide insights into the mechanisms of commensal-to-pathogen transitions and could lead to novel diagnostics and therapeutic interventions.

The highly diverse human-restricted species *Haemophilus influenzae* is a normal member of the upper airway microbiome, especially prevalent in the nasopharynx of healthy children [6,7]. Although infant mortality due to invasive infections was dramatically reduced by introduction of the conjugated vaccine that targets strains with the serotype *b* polysaccharide capsule, nontypeable *H. influenzae* (NTHi) strains continue to cause substantial morbidity. Specifically, NTHi are major contributors to exacerbations of chronic obstructive pulmonary disease (COPD) in older adults and middle-ear infections in children (or otitis media, OM) [8–11]. As the contribution of the pathogen *Streptococcus pneumoniae* to OM disease burden has been reduced over time by vaccines against the pneumococcal capsule, NTHi have become the most prevalent OM-associated bacteria [12–15]. More than half of children have an OM episode before 3 years of age, and up to a quarter go on to have recurrent or chronic OM. Although acute OM episodes often resolve on their own or with a course of antibiotics, recurrent and chronic cases can lead to delayed language development and sometimes permanent hearing loss. As a result, OM infections are a leading reason that children go to the doctor, are prescribed antibiotics, and receive surgery [16–19].

Whole-genome sequence analysis of *H. influenzae* has been ongoing since the laboratory strain Rd KW20 became the first cellular organism to have its genome completely sequenced in 1995 [20]. As with many other bacteria, subsequent genomic analysis of many strains with diverse provenance revealed the extent of species-level genomic diversity, not only due to allelic variation but also due to an extensive “pangenome”, in which only a fraction of genes are found across all strains of a species (“core” genes) and several fold more genes are found only in subsets of strains (“accessory” or “distributed” genes) [21–24]. *H. influenzae* is also naturally competent, with a DNA uptake apparatus biased towards “uptake signal sequence” (USS) motifs enriched in the genomes of *H. influenzae* and close relatives, thus natural competence and transformation results in frequent genetic recombination among strains [25]. This combined with observations of high diversity in gene content led to the “distributed genome hypothesis” and related models, which posit that the gain and loss of accessory genes into and out of strains by genetic recombination provides a mechanism for the rapid adaptation of *H. influenzae* and other species to different ecological contexts [26–30].

The high genomic diversity among bacterial strains of the same species is reflected in their highly variable phenotypes for traits including pathogenesis, cell surface epitopes, growth characteristics, intracellular invasion rates, serum resistance, antimicrobial resistance, biofilm formation, natural competence, virulence in animal models, and others (*e.g.,* [31–40]). Such data support a role for genetic variation in *H. influenzae* by modulating its pathogenic potential [41–43]. They also suggest that genome-wide association studies may identify genes or alleles with roles in causing disease. Evidence for contributions by accessory genes to pathogenesis has come from statistical genetic association studies of strains isolated from health and disease states, and this approach has previously identified novel virulence factors [23,44]. Other studies focused on COPD have used longitudinally collected strains to identify evolutionary adaptations of *H. influenzae* during long-term lung infection. These have identified recurring gene losses associated with survival in the COPD inflammatory environment, gene content differences, and recurring genomic rearrangements associated with long-term adaption to the COPD lung [45–48]. Past studies have also surveyed for associations of candidate genes or strain types with pediatric OM disease, but genome-wide association studies have only recently become practical. One recent study searched for differences between isolates from pneumonia and controls, identifying a strong association of disease with *H. influenzae* resistance to ß-lactam antibiotics [49]. Another recent study searched for signatures of recurrent evolution in isolates collected from 11 children who had OM with otorrhea (at 13 sampling points), finding evidence for point mutations in several genes, mostly associated with the cell wall biosynthesis or metabolism [50]. However, to our knowledge, no genome-scale studies associating *H. influenzae* genetic variation with human OM disease risk have been reported.

During OM, the middle ear microenvironment changes from an air-filled cavity to a fluid-filled one that contains planktonic and biofilm bacteria, infiltrating immune cells, and increased activity by specialized epithelial cells. The associated inflammatory processes are multifactorial with contributions from host cells especially neutrophils, viruses, and bacteria [51–53]. Notably, clinical studies have found that middle ear fluids can often be alkaline, in contrast with the acidic pus typically associated with purulent infections [54–58]. Laboratory studies further support links between alkalinity, OM severity, and biofilm formation [36,59], findings that raise questions about how host-microbe interactions modulate middle ear fluid composition and whether these changes affect OM burden or the risk of persistent disease.

To identify *H. influenzae* accessory genes associated with OM, we leveraged a large-scale long-term biorepository of prospectively and longitudinally collected bacterial strains isolated from healthy and sick children during the first three years of life in Rochester, NY, USA. This ongoing collection has been a crucial resource for epidemiological tracking of bacteria that cause OM, including population-level bacterial responses to new vaccine introductions [13,60–62]. The Rochester biorepository has also been crucial for identifying OM-prone children that experience recurring episodes of acute OM, at least in part due to delays in innate and adaptive immune development [63]. We retrospectively selected a set of >200 *H. influenzae* strains that were collected from 21 subjects persistently colonized by *H. influenzae* during early childhood, 12 of whom were OM-prone (experiencing recurring episodes of acute OM disease), performed a phylogeny-informed association study, and tested a top candidate operon for *in vitro* and *in vivo* phenotypes.

## Results

### Transient and persistent population dynamics of *Haemophilus influenzae* in healthy and om-prone pediatric cohorts

To examine the colonization and infection dynamics of *H. influenzae* within and among human subjects over time and to identify specific *H. influenzae* accessory genes associated with OM disease risk, we sequenced and analyzed the genomes of clinical isolates collected in Rochester, NY from the upper respiratory tracts of children during the first 3 years of life. Strains in this ongoing long-term large-scale biorepository are prospectively collected: multiple specimens from the nasal/nasopharyngeal tract (NP) are collected during 6 healthy pediatric visits between 6 and 36 months of age. In addition, during any acute OM episodes experienced by enrolled subjects, specimens are collected from both the NP and directly from middle-ear fluid (MEF) obtained by tympanocentesis.

We selected longitudinal series of isolates from the biorepository collected between 2008 and 2016 from 9 healthy and 12 OM-prone subjects. Selected series were chosen based on *H. influenzae* being isolated at ≥ 3 healthy visits for all subjects and at ≥ 4 sick visits for OM-prone subjects. We obtained 206 high-quality whole-genome shotgun assemblies from these isolates; all were sequenced using short-read Illumina technology, and a subset were also sequenced using long-read Pacific Biosciences technology. We next annotated their genes and clustered protein-coding genes based on homology to determine a matrix of gene presence/absence across isolates (Fig 1A; subject and genome information in S1 Table and S2 Table, respectively; the column dendrogram uses the phylogenetic tree shown in Fig 1B) [45,64]. Codon-aware nucleotide alignments of single-copy genes were used to produce a phylogenetic tree modeling the population structure of the isolate collection (Fig 1B, S1 File) [45,65,66]. Finally, to track distinct strains within and among subjects over time, highly related isolates were grouped into 63 distinct “clonal lineages” (CLs) using PopPunk [67] (Fig 1C). Other methods to define CLs gave nearly identical groupings (S1 Table). Notably, although many CLs were sampled more than once (48 of the 63 CLs), the overall genetic diversity captured in this isolate set spanned species-level diversity, as illustrated by a phylogeny with 39 complete genomes from this study together with 38 complete genomes retrieved from RefSeq collected from diverse sources (S1 Fig).

**Fig 1:**
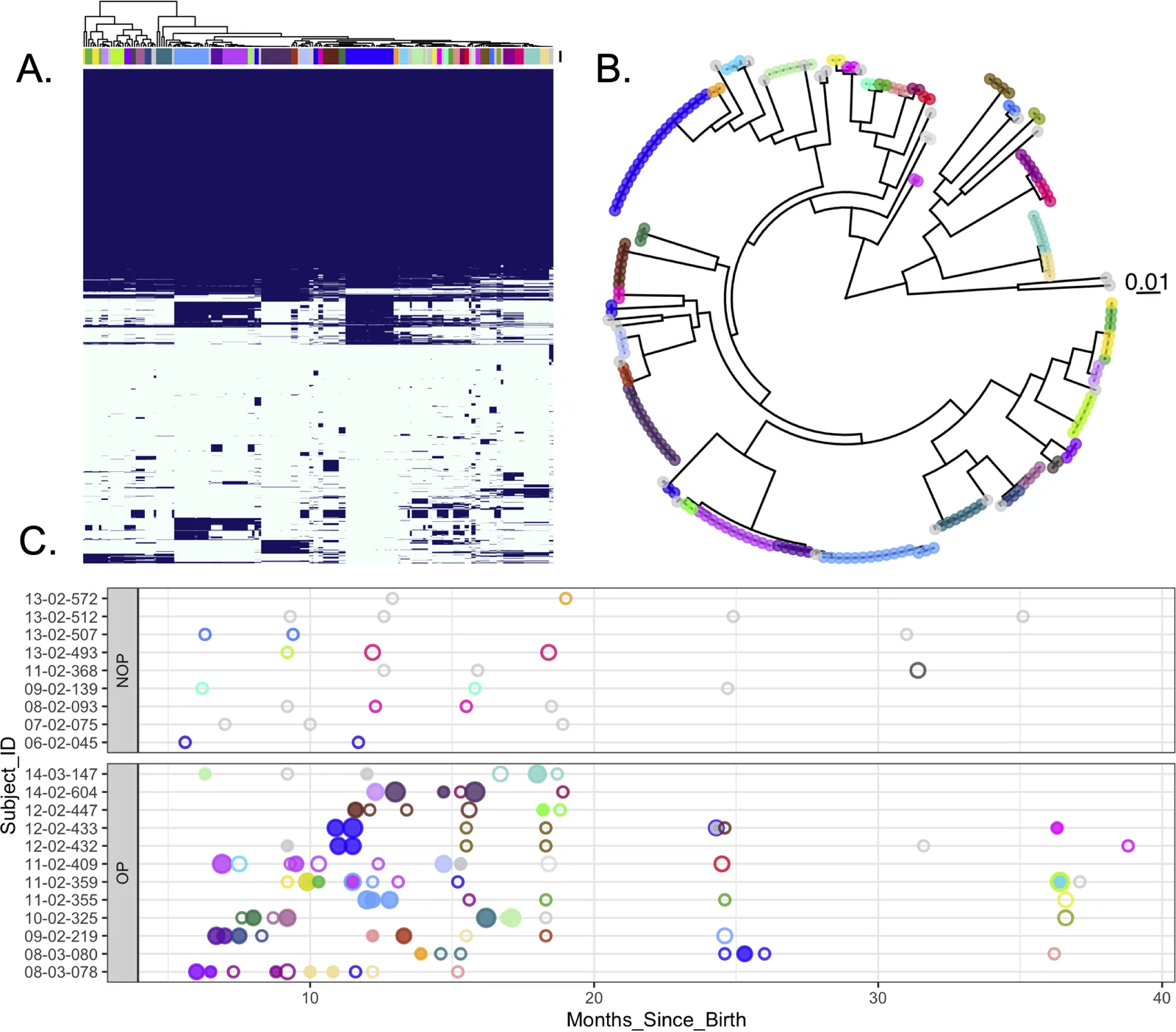
Colonization and infection over time by distinct clonal lineages (CLs) in 9 healthy and 12 OM-prone children. **A.** Presence/absence of homologous protein-coding gene clusters (rows) in the genomes of *H. influenzae* isolates collected from healthy and OM-prone children (columns). Dark blue indicates gene presence; pale blue indicates absence. Rows were hierarchically clustered to group genes with similar distributions across isolates, and the column dendrogram uses the species-level phylogeny shown in Fig 1B. **B.** Phylogenetic tree inferred from 3013 single-copy gene alignments (1315 of them core). Closely related isolates grouped into clonal lineages (CLs) have the same tip color, except for those labeled in light grey, which indicates singlet lineages that appeared only a single time. **C.** Colonization and infection dynamics in two cohorts (NOP: not OM-prone; OP: OM-prone) with each row indicating a single subject and circles indicating doctor’s visits from which *H. influenzae* was isolated. Filled circles correspond to isolation at OM episodes, and open circles indicate a scheduled pediatric check-up; distinct colors correspond to distinct CLs (except light grey), as described in **B**; the size of each circle is proportional to the number of distinct isolates (from 1 to 6) collected from each sampling point.

The pattern of distinct CLs seen within and among subjects over time illustrates the complexity of *H. influenzae* population dynamics, showing evidence of transience, persistence`, and recurrent colonization and infection (Fig 1C, S1 Table). The top-3 most-abundant CLs were biased to OM visits (45 of 50 isolates in the top-3 CLs were from OM) whereas “transient” CLs observed at only one visit were biased towards healthy visits (only 3 of 12 singlet CLs were from OM). However, many CLs were found at both healthy and OM visits, and many isolates from the same CL were often sampled from the same subjects and time points, particularly during OM. These results indicate that OM-causing isolates are not restricted to specific branches of the *H. influenzae* phylogenetic tree. Although it is possible that some lineages may be more prone to causing OM than others, because many CLs were re-sampled from OM-prone children, this could also simply be a property of the study design.

Since more than one isolate was often sequenced from a single subject and time point (from 2 to 8 isolates at 51 of 120 visits), we evaluated the rate of polyclonal colonization by multiple strains, albeit with a high limit of detection. This found that in 11.8% of visits with multiple isolates, more than one lineage was identified (6 of 51 visits had > 1 CL collected from distinct specimens). Thus, single CLs tended to predominate within time point and subject, even though distinct CLs had variable persistence over time. These patterns corroborate previous analyses of longitudinal series collected from health and disease, as well as studies that show middle ear fluid (MEF) isolates frequently closely match isolates from the nasopharynx and nasal passages (NP) that were collected during the same OM episode (~ 85%) [45,46,62,68–70].

Co-colonization and co-infection with *H. influenzae* by other bacteria could be partially assessed, since other OM-associated species were routinely isolated and cultured during specimen collection (Fig 1, S2 Table), particularly the other two common OM pathogens *S. pneumoniae* and *Moraxella catarrhalis*. Isolation of *H. influenzae* without any other species was more frequent during OM than at healthy visits (55.7% of OM visits *vs.* 16.1% of healthy visits, Fisher test p-value < 0.01). Similarly, isolation of *H. influenzae* by itself was higher in MEF over NP specimens (84.5% of MEF samples *vs.* 16.0% of NP samples, p-value < 0.01). This effect was comparable when healthy visits were excluded (15.6% of NP samples collected during OM, p-value < 0.01). Collectively, these data show that although polymicrobial colonization of the NP is common, OM infections by *H. influenzae* are often predominated by single strains within both the ME and NP, consistent with other studies.

### Association testing between OM disease and *H. influenzae* accessory genes

Because the scope of genomic variation among clinical isolates is extensive but the set of genomes sequenced here was relatively small, we focused only on whether the presence or absence of annotated genes/operons was associated with OM. Although this likely missed important associations with specific alleles and may have suffered from artifacts of gene annotation and homolog clustering, it drastically reduced the number of statistical tests, simultaneously enabling focus on the predicted molecular functions of the genes identified. In the set of 206 genome assemblies, 1315 of 3267 were core genes, with one or more homologs found across isolates (Fig 1A, see legend). The 1951 accessory genes that remained could be further collapsed into 724 ‘segregation patterns’ or ‘features’, since many accessory genes were identically distributed across all 206 genomes (typically due to co-inheritance as operons). Focusing on these 724 accessory gene features thus excluded the many thousands of single-nucleotide, indel, and more complex genetic variants distinguishing these isolates, in favor of association testing with a reduced set of binary protein-coding gene presence/absence encodings.

To test for associations between accessory gene features and OM disease, we used the Scoary pipeline [71]. First, we applied Fisher’s Exact Test to identify accessory genes with skewed isolation from MEF compared to NP. Next, to account for phylogenetic dependencies that might spuriously associate markers with OM-associated clades, we tested for evidence of multiple independent ancestral gene-trait co-occurrences across the phylogeny [71–73]. **Table 1** lists the seven accessory gene features most strongly associated with isolation from the middle ear. Although many isolates were clustered into clonal lineages (often from the same subject or sampling point), the top candidate associations did not show simple statistical associations with health or disease states, particular subjects, or clonal lineages. Instead, distinct evolutionary histories were observed for each candidate, in which recurring gene acquisition and/or loss along the phylogenetic tree correlated with gains and/or losses of health or disease states at the tips of the tree. These results suggest that the presence of these accessory genes could directly affect the risk of OM infection.

**Table 1:** Associations between accessory gene features and isolation from middle-ear fluid (MEF) *vs.* nasopharynx/nasal passages (NP) by naïve Fisher’s exact test with a Benjamini-Hochberg adjusted p-value < 0.05 (FDR), as well as a “best” p-value for phylogenetic gene-trait co-occurrence of < 0.05 (Pairwise Comparisons – Best) from Scoary. All MEF isolates were collected from subjects during acute OM episodes, but NP isolates come from both health and OM disease. The numbers in parentheses indicate the subset of NP isolates collected during OM episodes instead of healthy visits. Further details are in S3 Table, including for two other clinical “traits”: whether isolated from an OM *versus* healthy visits or (b) whether from an OM-prone *versus* healthy subject. Only genes/operons that passed both the FDR-adjusted Fisher’s test and “best” comparisons of phylogenetic contrast at p < 0.05 were included. Genes strictly co-inherited across strains were treated as single tests and are separated by the double dashes (--).

	Middle Ear Fluid		Nasal/Throat/ Nasopharynx				Pairwise Comparisons	
Gene/Operon	Present	Absent	Present (OM)	Absent (OM)	Log_2_(Odds)	FDR	Best	Worst
*potE--speF*	2	66	42 (6)	96 (60)	-3.85	0.0003	0.031	0.219
cluster_1982	3	65	41 (8)	97 (58)	-3.20	0.0009	0.016	0.453
*toxT*	63	5	95 (52)	43 (14)	2.51	0.0029	0.021	0.344
*iciA--cadA*	0	68	21 (3)	117 (63)	-Inf	0.0046	0.031	0.031
*allC*	4	64	35 (3)	103 (63)	-2.44	0.0092	0.031	0.688
*pyrG* *_2*	5	63	32 (8)	106 (58)	-1.93	0.0422	0.031	0.688
cluster_1202	63	5	106 (58)	32 (8)	1.93	0.0422	0.031	0.688

### The absence of *H. influenzae* polyamine metabolism genes is associated with isolation from OM disease states

Notably, of the seven associations, five were “anti-OM” associations, where the presence of the gene or operon correlated with healthy carriage rather than disease (*i.e.,* the associations with negative log_2_(odds ratios) in **Table 1**). One positive association with OM was to an accessory gene *toxT* predicted to encode the toxin in a recently characterized competence-regulated toxin-antitoxin system [74]. This putative “pro-OM” gene would be an interesting candidate for future follow up studies on its potential role in pathogenesis, but we noted that most candidates were “anti-OM” genes/operons, each with a plausible role in nitrogen/polyamine metabolism and pH homeostasis:

(1) The top candidate, *potE-speF*, is further investigated below (Table 1, row 1). Evidence for a genetic association of *potE-speF* absence and OM disease comes from the nearly 16-fold depletion of this operon in MEF compared to NP isolates (Fig 2A; Table 1, row 1), as well as a history of gains and losses that correlates with changes in health status at the tips of the phylogeny. A parsimonious model of *potE-speF* acquisition in seven independent lineages would suggest that five are associated with loss of virulence in several different subjects (Fig 2B). The presence-absence calls of these two genes were not an artifact of annotation or clustering method, since all copies had > 95% amino acid identity, tblastn found no partial copies on the genome assemblies, and the local gene neighborhood was consistent in strains that lacked *potE-speF* (S2 Fig). The *potE-speF* operon is predicted to encode machinery that exchanges and interconverts polyamines with the extracellular environment: *potE* encodes a predicted inner membrane putrescine-ornithine antiporter that exchanges neutral extracellular ornithine for alkaline cytosolic putrescine, and *speF* encodes a cytosolic ornithine decarboxylase converting ornithine into putrescine (Fig 2C). In *E. coli*, the orthologous *potE-speF* operon is known to confer ornithine-dependent acid tolerance, since the neutral polyamine ornithine is converted into weakly basic polyamine putrescine, thereby allowing *E. coli* to directly neutralize an acidic environment when in the presence of ornithine [75–78].

**Fig 2:**
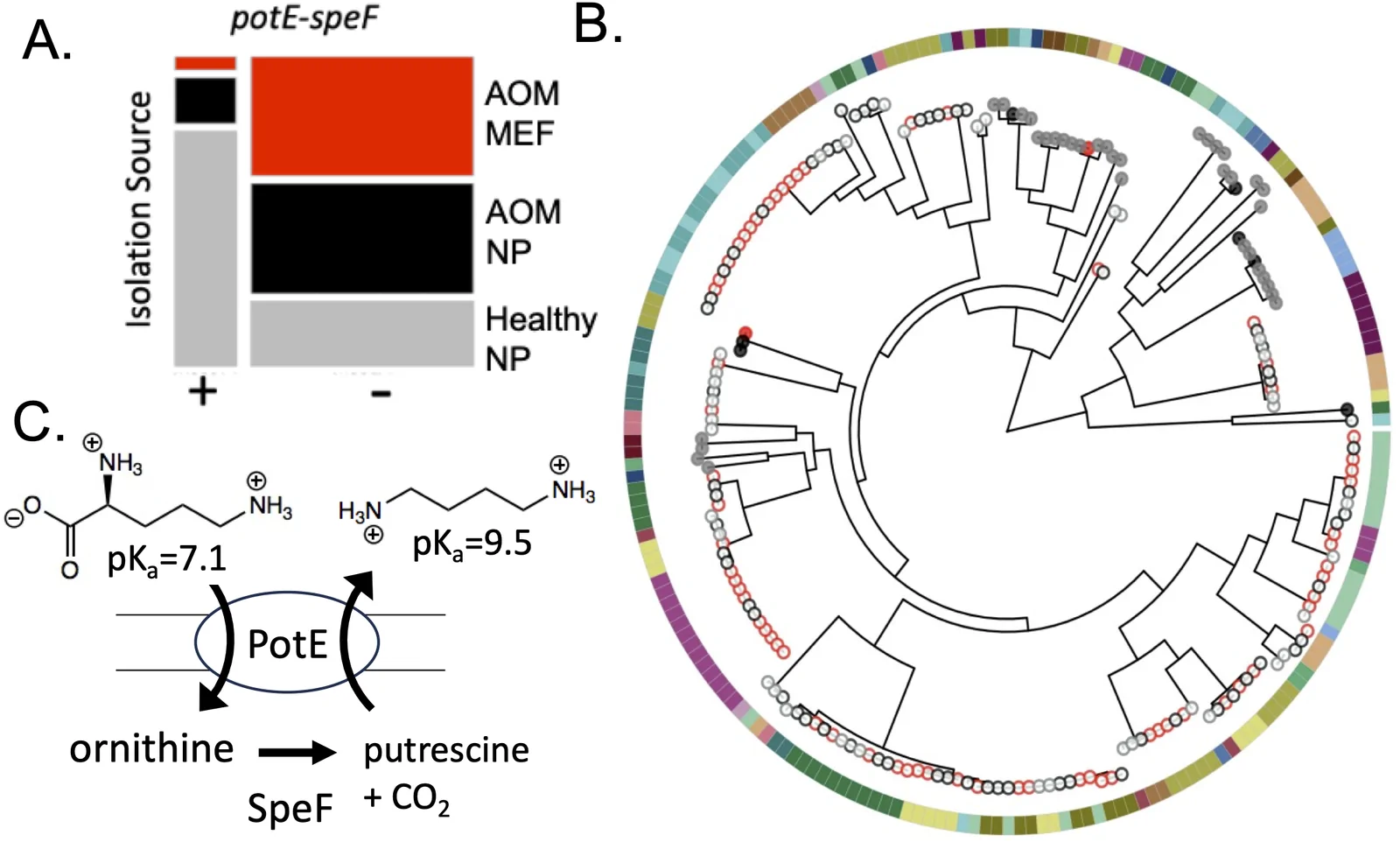
*H. influenzae* accessory operon *potE-speF* is negatively associated with otitis media. **A.** Mosaic plot showing the distribution of *potE-speF* operons across isolates. AOM = acute otitis media; MEF = middle-ear fluid; NP = nasal or nasopharyngeal. **B.** Phylogenetic tree has the same topology as Fig 1B and S1 File: filled tips indicate presence of *potE-speF* and open tips indicate absence; tip colors indicate isolation source as in mosaic plot, red = isolation from a subject with AOM from MEF, black = isolation from a subject with AOM from NP, light grey = isolation from the NP of a subject without AOM; distinct outer ring colors indicate isolation from distinct subjects. **C.** Diagram of predicted functions of PotE and SpeF proteins. PotE acts to take up neutral extracellular ornithine and secrete alkaline intracellular putrescine, while SpeF decarboxylates ornithine into putrescine.

(2) A putative cyclase-encoding candidate gene, “cluster_1982”, was nearly always co-inherited with *potE-speF* though not as part of the same operon or in the same gene neighborhood. The cluster_1982 gene was absent from only a single isolate with *potE-speF* and was present in only one without it (**Table 1**, row 2). Thus, this association with OM is highly correlated with *potE-speF*. No further information about potential biochemical functions was found.
(3) The *iciA-cadA* candidate operon was identified in 10.1% of all isolates but only 1.4% of OM isolates and none of the MEF isolates (Table 1, row 4). The canonical *iciA* gene from *E. coli* encodes a transcriptional regulator, and the canonical *cadA* encodes lysine decarboxylase, which converts lysine into the polyamine cadaverine (weakly basic) while consuming a proton and releasing CO_2_ [78–82]. Like *potE-speF*, these genes have been implicated in both pH homeostasis and pathogenesis, and their activity impinges on central metabolism through their interaction with the tricarboxylic acid pathway [83,84].
(4) The *allC* accessory gene is predicted to encode an allantoate amidohydrolase, which breaks down the tetraamine allantoate into the triamine S-ureidoglycolate, consuming protons and water, while releasing carbon dioxide and ammonium ion (weakly acidic). In *E. coli*, the *allC* gene product is involved in the breakdown of purines for anaerobic nitrogen utilization [85,86]. Although we found no specific reports demonstrating a role in pH homeostasis or pathogenesis, *allC* was found in 19.2% of all isolates but only 5.9% of OM isolates, with a relative depletion of nearly 13-fold in MEF compared to NP isolates (Table 1, row 5).
(5) Finally, a diverged paralogous copy of *pyrG* (*pyrG_2*) (Table 1, row 6) encodes a putative CTP synthetase, which in *E. coli* converts UTP to CTP, consuming an ATP and using glutamine or ammonia as a nitrogen source. Notably, whenever this candidate was present, an unannotated accessory gene cluster_1202 was absent (Table 1, row 7), and vice versa, so these two accessory genes are mutually exclusive and have reciprocal distributions across isolates. No predicted functions could be assigned to cluster_1202 so this may be an additional “pro-OM” gene, but the *pyrG_2* gene, like other “anti-OM” candidates, could plausibly affect nitrogen cycling, pH homeostasis, or have other direct or indirect effects on central metabolism.

### Candidate “Anti-OM” Operon *potE-speF* confers ornithine-dependent alkaline sensitivity

Taken together, all five strong “anti-OM” associations had predicted molecular functions in similar or related pathways (**Table 1**). This suggests that natural bacterial genetic variation in polyamine metabolism and pH homeostasis affects the risk of middle-ear disease. Several candidates have known or plausible roles in the ability of bacteria to survive acid stress, whereas reports in the literature have indicated that middle-ear fluids from OM can sometimes have an alkaline pH, instead of the acidic pH often associated with other purulent secretions [54–58]. In particular, the predicted molecular functions of genes in the top candidate accessory operon *potE-speF* (Fig 2C) led us to hypothesize that the absence of *potE-speF* increases bacterial survival in an alkaline environment. To test this, we evaluated growth and survival of a wild-type (WT) strain (the low-virulence laboratory strain Rd KW20, which naturally possesses *potE-speF*) and a mutant derivative carrying a *∆potE ∆speF* double knock-out mutation. During strain construction, a *∆potE* single mutant was also produced, but the *∆speF* single knock-out was unable to be generated, suggesting that this gene may be conditionally essential when *potE* is present. To identify genetic differences between WT and *∆potE ∆speF* outside the knockout constructs that could have phenotypic effects, we used high-coverage genome sequencing. This found many sites with high within-strain genetic heterogeneity (*i.e.,* 222 sites had 5–25% variant allele frequencies within both cultures), reflecting the many phase-variable simple sequence repeats (SSRs) in the *H. influenzae* genome (and possibly some persistent sequencing artifacts). Only five positions had asymmetric allele frequency distributions between WT and *∆potE ∆speF* samples, but in all five cases, none was a fixed difference that distinguished the strains (S4 Table). Thus, phenotypic effects of these putative genetically unstable sites would affect both strains. Experiments reported here focused attention on comparing the WT and *∆potE ∆speF* double mutant strain, since both genes were always both present or absent across the set of genomes analyzed.

Since we hypothesized that the presence of *potE-speF* would lead to ornithine-dependent effects on growth and survival through effects on pH, we first measured differences between WT and mutant growth curves (changes in OD_600_ over time) for cells in rich media adjusted to three different initial pHs (6.0, 7.5, or 9.0), with ornithine supplementation at four concentrations (0, 12.5, 25, or 50 mM) across a physiologically relevant range [83]. Representative shaking culture growth curves are shown in Fig 3A and areas-under-the-curves are summarized in Fig 3B. All culture conditions supported growth over time, but WT cells grown with high concentrations of ornithine showed an initial extended exponential phase, followed by decreased OD_600_ after the cultures entered stationary phase, potentially indicative of cell death and lysis. Furthermore, this effect was more pronounced at higher pHs (Fig 3A, pink lines; Fig 3B, blue lines). By contrast, the *∆potE ∆speF* double mutant showed no ornithine-dependent differences in growth at all three pHs (Fig 3A and Fig 3B, lower panels), consistent with a requirement for these genes in converting neutral ornithine in the extracellular environment into putrescine.

**Fig 3:**
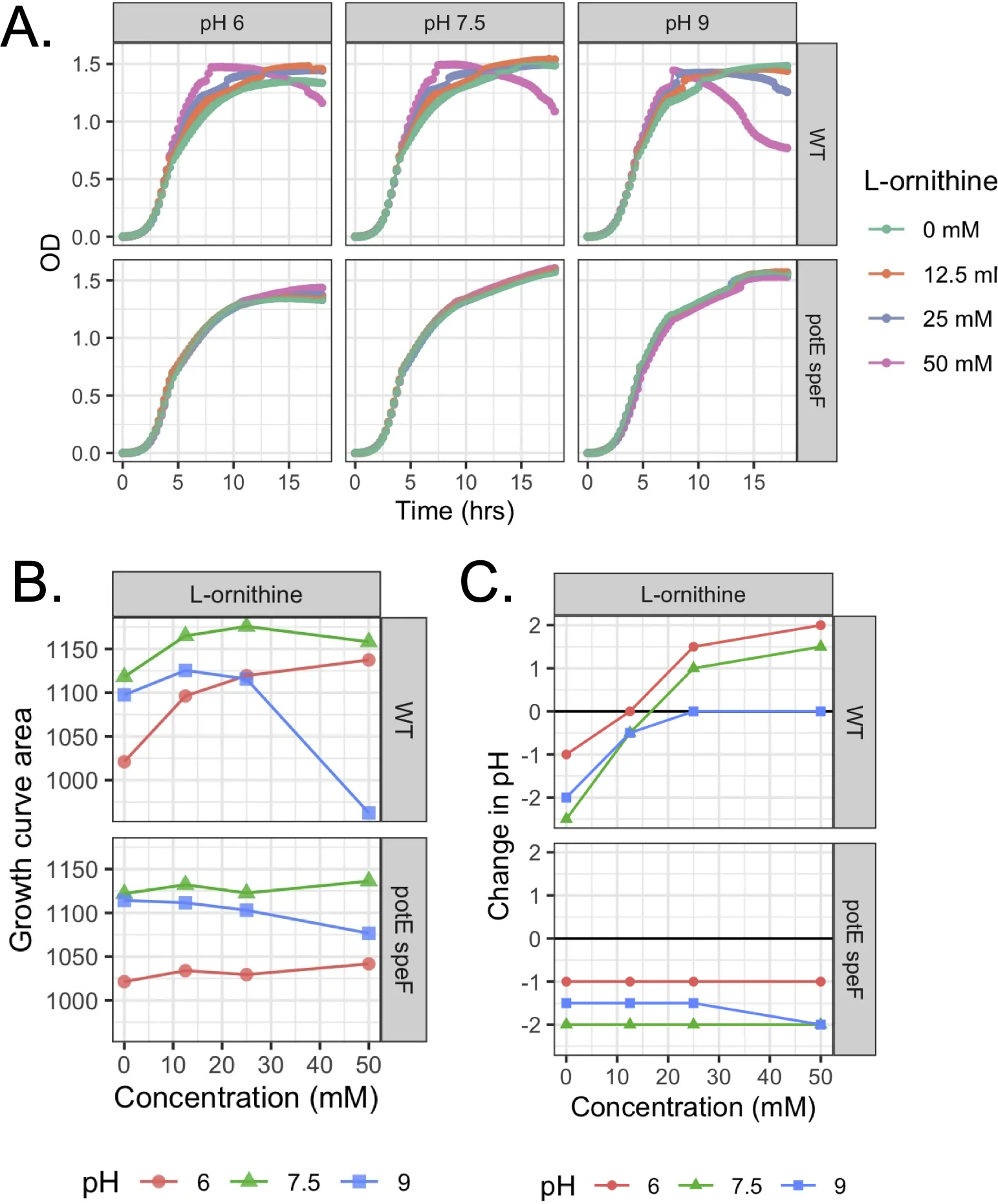
Effect of *∆potE* *∆speF* on planktonic growth in shaking culture; representative experiment shown. **A.** Growth over time for subcultures measured by OD_600_ every 15 min over 16 h in WT and *∆potE ∆speF* strains (rows), at 3 initial pHs (columns), and 4 ornithine concentrations (colors). **B.** Areas-under-the curve (y-axis) with varying ornithine (x-axis) at three initial pHs (colors) and two genotypes (panels). **C.** Changes in culture pH after overnight growth.

Measurements of culture pH after overnight growth found a strong effect of *potE-speF* on final pH when in the presence of ornithine (Fig 3C). As expected, cultures of both genotypes without supplementation became more acidic overnight, due to the accumulation of acidic byproducts of primary metabolism in the medium, regardless of initial pH. With ornithine supplementation, WT cultures showed concentration-dependent increases in the final pH overnight, as predicted. By contrast, deletion of *potE-speF* showed insensitivity to supplemental ornithine, and so all cultures at all starting pHs showed comparable pH decreases towards acidity during overnight growth.

In parallel, we examined how supplementation with six other polyamines that had previously been detected in *Haemophilus* [87] affected the growth of WT and of *∆potE ∆speF* mutant strains. In addition to ornithine, we also tested cadaverine, diaminopropanol, putrescine, spermidine, spermine, and tyramine. Supplementation with all tested polyamines had distinct pH-specific effects on growth but none showed a strong difference between WT and *∆potE ∆speF*, except ornithine and (weakly) putrescine (S3, S4 and S5 Figs). These results show that polyamine content and the pH of the environment have strong effects in *H. influenzae* growth and show a specific relationship between *potE-speF* activity and growth in the presence of ornithine or putrescine.

Since the growth curves in Fig 3A used OD_600_ biomass measurements and so did not directly measure viability, we next measured CFU/ml titers after 18 h overnight growth (Fig 4). In the absence of supplemented ornithine, both wild type and *∆potE ∆speF* showed significantly and substantially higher titers (>30-fold) after overnight growth when inoculated into medium with a higher initial pH (mean 7.5x10^7^ CFU/ml at starting pH 6; 6.8x10^8^ at pH 7.5; and 2.8x10^9^ at pH 9; two-way ANOVA: p-value < 10^-5^ for pH effect; p-value = 0.51 for genotype effect). The increased survival of cells in overnight cultures at higher pHs is likely because acidic waste products of metabolism were more effectively neutralized. By contrast, supplementation with ornithine (50 mM) had strong genotype-dependent effects on survival at the neutral and alkaline pHs. No detectable CFU were seen for wild type overnight grown with added ornithine (limit-of-detection <10^2^ CFU/ml), but the *∆potE ∆speF* mutant remained viable with supplemented ornithine (mean 2.4x10^8^ CFU/ml for starting pH 7.5; 1.5x10^9^ for pH 9). At both pH 7.5 and pH 9, two-way ANOVA found significant effects of ornithine supplementation (p-value < 10^-4^). Although no effect of genotype alone was seen (p-value > 0.7), a strong genotype-by-treatment interaction was observed (p-value < 10^-3^). At a starting pH 6, no effects were seen by two-way ANOVA, however overnight viability of cells in media at starting pH 6 was substantially lower than for higher pHs with some titers below the limit-of-detection (Fig 4, downward triangles).

**Fig 4:**
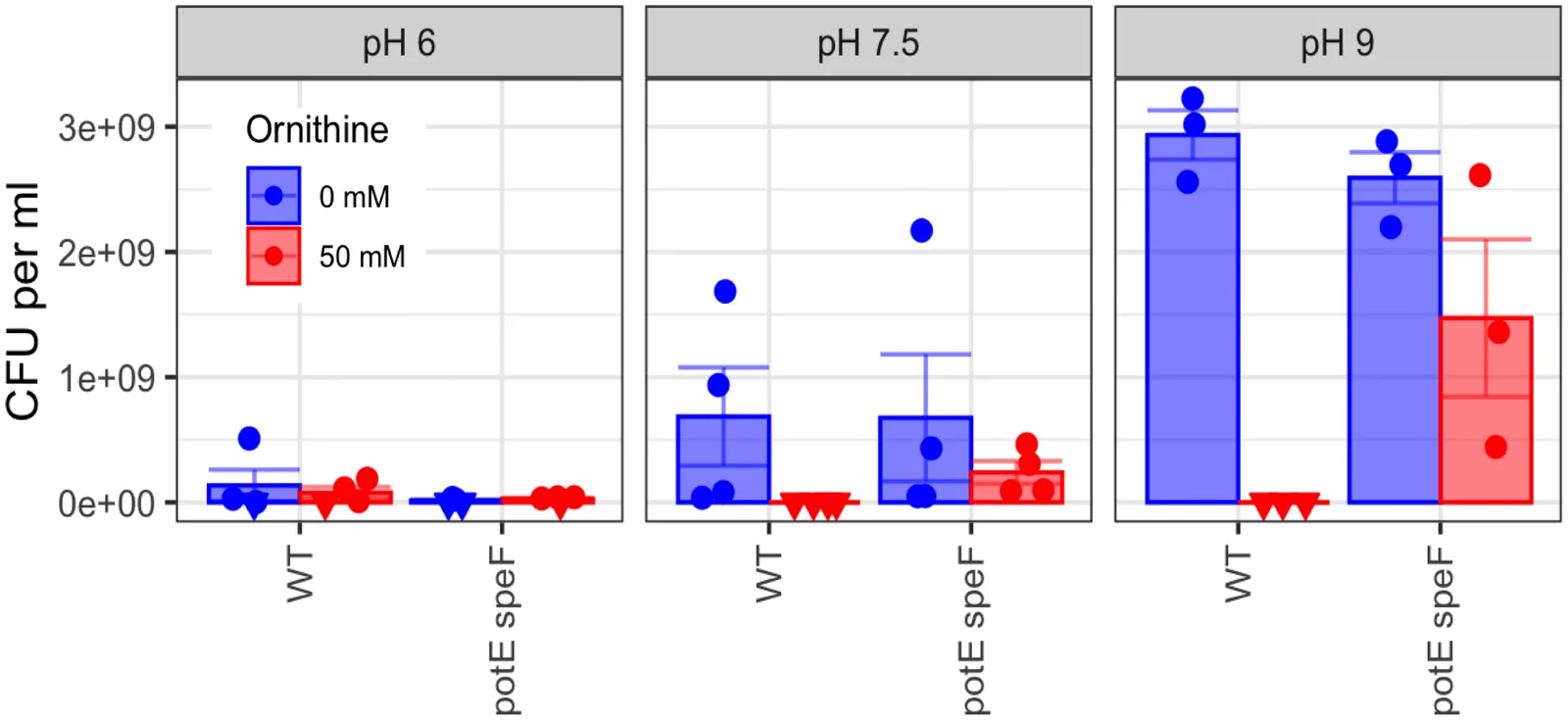
Ornithine-dependent cell death in alkaline conditions is bypassed by deletion of *potE-speF.* Cultures were grown in sBHI medium with 0 (blue) or 50 mM (red) supplemented L-ornithine at three different initial pHs for 18 h before dilution and plating to sBHI agar plates for colony counts and CFU/ml calculations. Independent replicates were carried out on separate days with independent cultures; circles indicate CFU/ml titers and downward triangles indicate cultures with no detected viable CFU at a limit-of-detection of ~10^2^ CFU/ml. Boxes indicate the mean, and error bars indicate the standard deviation.

These results show a strong ornithine-dependent viability defect in WT cells when grown in media starting at neutral or alkaline pH. This viability defect depends on the presence of *potE-speF*, since deletion of the operon bypasses ornithine-dependent cell death at high pH. No significant difference in acid tolerance was observed between genotypes as previously reported for *E. coli* [75–78], possibly reflecting the particulars of these assays and/or organism-specific differences. As a notable aside, these results also show substantially higher survival of *H. influenzae* cells after overnight culture when inoculated into standard rich media adjusted to a high pH, which has practical implications for working with *H. influenzae* strains, which can often have low viable CFU/ml after overnight culture.

### The molecular functions of *potE-speF* confirmed by mass spectrometry

We next sought to confirm that the phenotypic effects observed for *in vitro* cultures were associated with the predicted function of the *potE-speF* operon to take up extracellular ornithine, convert it into putrescine, and secrete putrescine. We used liquid chromatography mass spectrometry (LCMS) to determine the relative quantities of different polyamines in cell pellets and lyophilized media supernatants of WT and mutant cultures grown at three pHs, with or without supplemented ornithine. Results after subculture for 5 h at different starting pHs and supplements are shown in Fig 5 (time points at 2, 4, 5, and 24 h are shown in S6 Fig). In the absence of supplemented ornithine, ornithine levels in both pellets and supernatants were low after 5 h for both genotypes at all three pHs (Fig 5A, top panels). With supplementation, ornithine levels remained low, except for cell pellets of the *∆potE ∆speF* mutant, which accumulated ornithine to high levels (>10-fold; ANOVA p < 10^-5^ for an ornithine-by-genotype interaction; p > 0.5 for effect of pH). These results are consistent with the known function of *speF*, and they further indicate that ornithine uptake does not necessarily require *potE*, likely instead supported by the partially redundant functions of the *potABCD* transporter or similar uptake systems.

**Fig 5:**
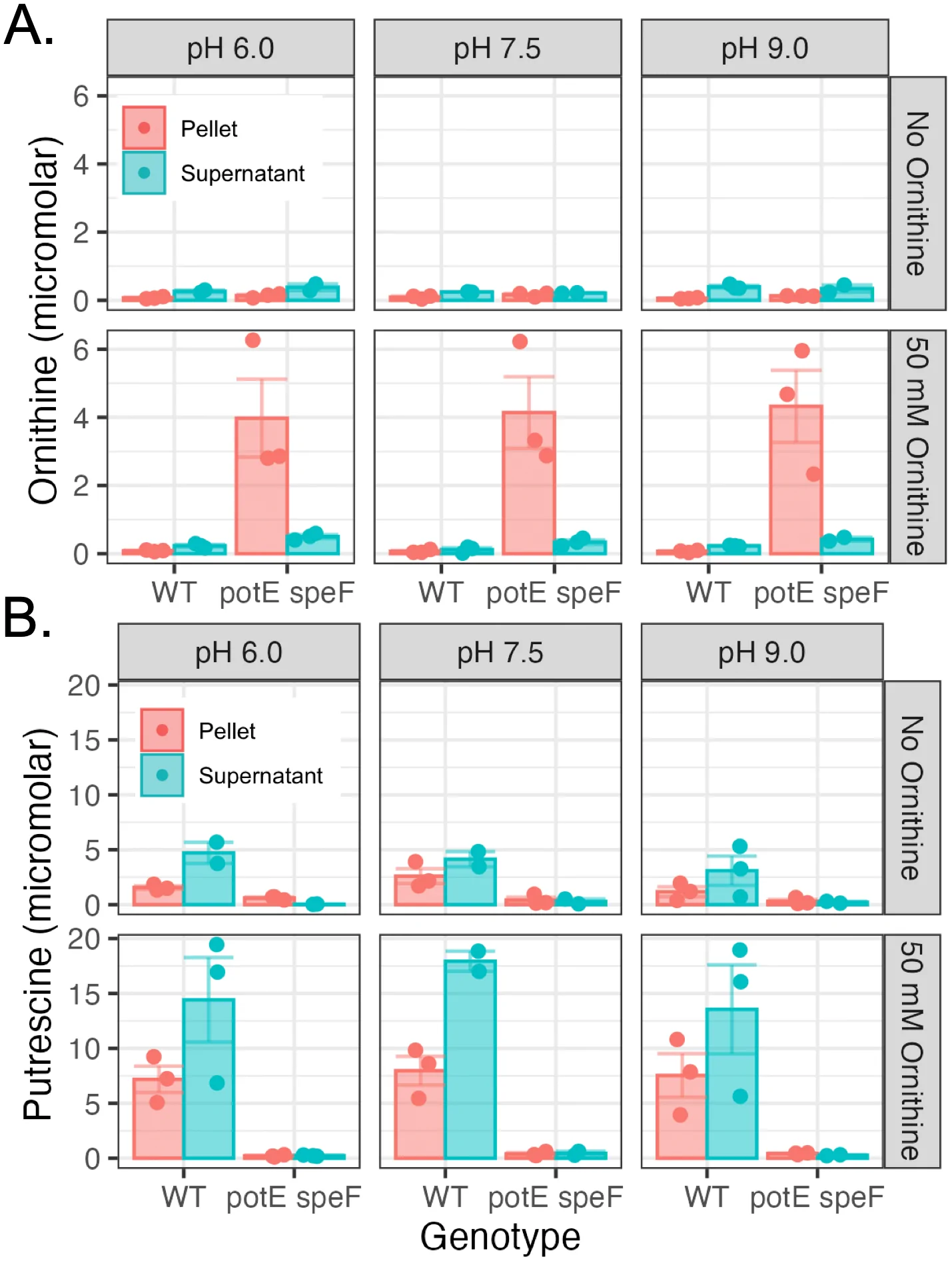
Mass spectrometric analysis of intra- and extracellular ornithine (A) and putrescine (B) levels in *H. influenzae* WT and *∆potE* *∆speF* mutants grown at different pHs, with or without supplemented ornithine (50 mM), after 5 h of growth. Cell pellets and lyophilized culture supernatants were derivatized and extracted before LCMS to quantify relative polyamine levels. The y-axis indicates the micromolar (µM) concentration of each polyamine in culture-equivalent volumes for the extracellular environment (supernatant) and cells (pellet). Results shown are for three independent replicates conducted on different days. Boxes indicate the mean, and error bars indicate the standard deviation.

Consistent with the predicted function of *speF*, putrescine levels for *∆potE ∆speF* were low in all conditions tested, indicating that putrescine production depends upon *potE-speF.* In further support of the predicted functions of the operon, in WT cultures, ornithine supplementation led to a > 2-fold increase in intra- and extracellular putrescine levels (Fig 5B, bottom *vs.* top panels, p = 1.1x10^-5^ by ANOVA; all time points shown in S7 Fig). In summary, ornithine can still be taken up in the absence of *potE*, as expected, but putrescine levels depend on *speF*. Notably, the starting pH had little overall effect on the transport and interconversion of ornithine and putrescine by cells. In conclusion, *potE-speF* causes ornithine-dependent production and secretion of putrescine, leading to increased media pH and survival defects of *H. influenzae* cells associated with alkalinity.

### The effects of *potE-speF* on other polyamines

In parallel, LCMS was also used to examine the relative levels of other polyamines previously detected in *Haemophilus* as part of a survey for polyamine markers that distinguish members of the *Pasteurellaceae* family [87] (S8 to S12 Figs). The relative abundances of spermidine, spermine, diaminopropanol and tyramine were unaffected by the *∆potE ∆speF* mutation; however, cadaverine showed markedly decreased levels in the absence of *potE-speF*, indicating that cadaverine levels are directly or indirectly affected by *potE-speF* activity (S8 Fig). These results show that the *potE-speF* gene products affect the level of metabolite(s) other than ornithine and putrescine.

### Inactivation of *potE-speF* increases disease burden in a mouse model of otitis media but not lung infection

Our genetic association study found that *H. influenzae* strains lacking *potE-speF* are enriched in OM disease, and *in vitro* growth assays showed that *potE-speF* confers ornithine-dependent sensitivity to alkalinity likely due to the production of alkaline putrescine. However, these data alone do not show a direct role for these genes in modulating OM pathogenesis. To test for differences in pathogen burden by WT and *∆potE ∆speF* mutant strains during infections, we employed two established mouse models of *H. influenzae in vivo* fitness, one for acute middle-ear infection after influenza viral infection, and another for lung infection [88,89].

To test whether the *potE-speF* genes affect OM disease burden, wild-type and mutant strains were tested in a mouse model of otitis media, in which mice are infected with influenza virus a week prior to intranasal inoculation of bacteria. Three days post-infection, burden was evaluated from nasal lavage, ME washes, and ME bullae. The wild-type Rd KW20 strain had a low bacterial burden in all three specimen types (~10^4^ to 10^5^ CFU), whereas the *∆potE ∆speF* mutant showed substantial and significant increases in bacterial burden (Fig 6A; ~ 32-fold for nasal lavage, ~ 63-fold for ME washes, and 6.2-fold for homogenized ME bullae, p < 0.05 for all sites or each site by one-way ANOVA on log-transformed CFU). These results indicate that the presence of the *potE-speF* operon limits the ability of *H. influenzae* to cause middle-ear disease after influenza viral infection.

**Fig 6:**
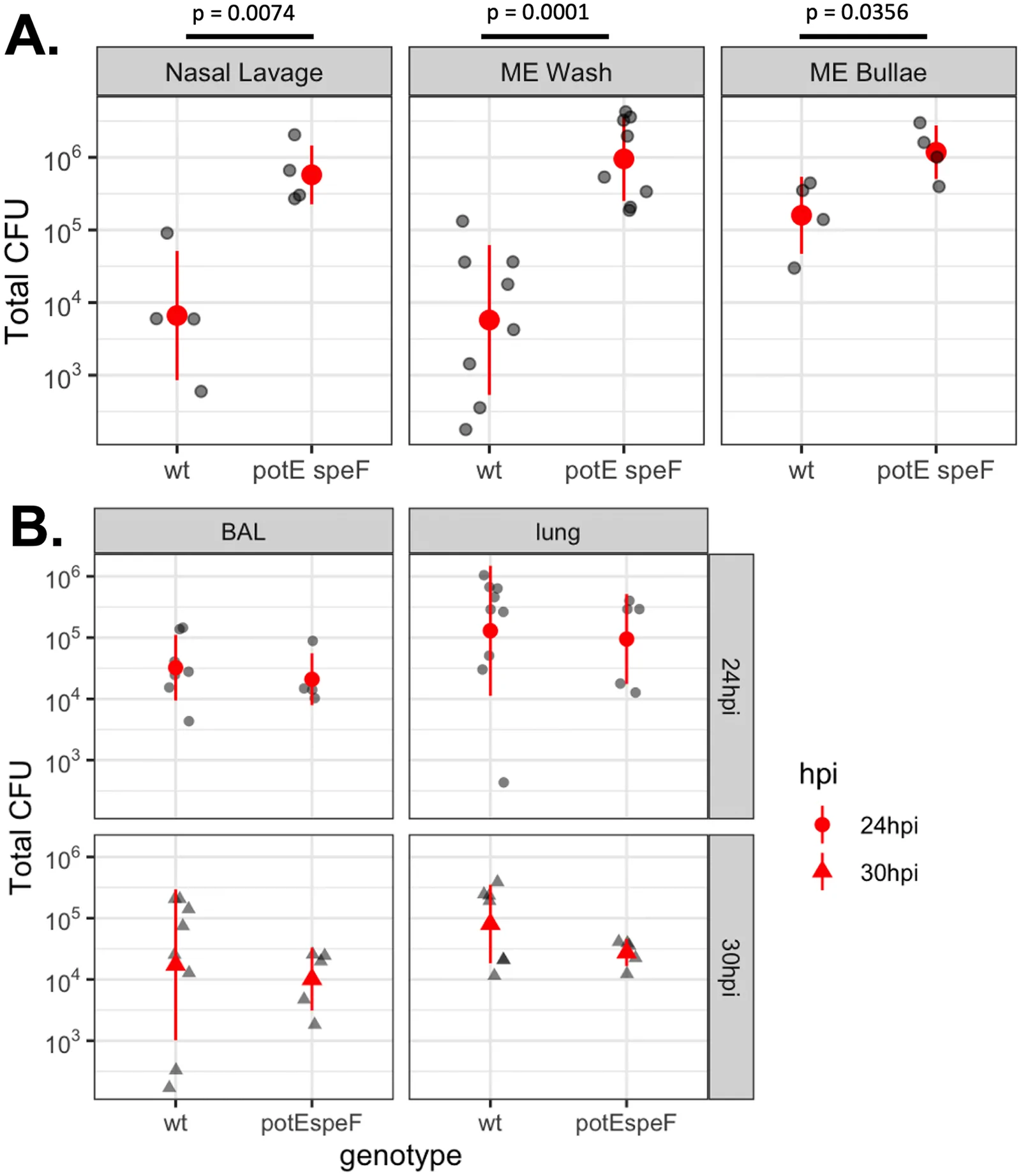
Mouse disease burden in middle ear and lung infection models. **A.** Infection of WT Rd and *∆potE ∆speF* mutants in a C57BL/6 mouse OM model. Three specimen types were recovered 3-days after intranasal inoculation of 10^5^ CFU into adult animals that were pre-infected with influenza virus 7-days earlier. Infectious burden was measured as total CFU recovered from each specimen type (n = 4 animals per genotype), plotted on a log-scale. ME washes were recovered from both ears separately resulting in 2 washes per mouse; ME bullae were pooled before homogenization. **B.** Infection of WT and *∆potE ∆speF* mutant strains in a CD1 mouse model of lung infection, in which animals (n ≥ 5 per genotype) were intranasally inoculated with 10^8^ CFU. Two specimen types were collected for dilution and plating to measure bacterial burden 24 or 30 h later, Bronchoalveolar lavage fluid (BALF) and homogenized lungs. Infectious burden was measured as total CFU recovered from each specimen type. Boxes indicate the mean, and error bars indicate the standard deviation. Statistically significant differences were evaluated using one-way or multi-factor ANOVA on log-transformed CFU. Significant p-values < 0.05 are shown.

Results from the OM model contrast with results from the lung infection model (Fig 6B). CD1 mice were intranasally inoculated with ~10^8^ CFU of WT or mutant bacteria. Lung disease burden after 24 or 30 h was evaluated by recovering bacteria from bronchoalveolar lavage fluid samples (BALF) or from homogenized lungs, diluting, and plating to sBHI agar solid media to determine total CFU counts per BALF and per lung. At 24- and 30-h post-infection, CFU titers were >100-fold lower than the original inoculum, but no significant differences were seen between clearance of the WT and *∆potE ∆speF* strains (multi-factor ANOVA p-value>> 0.05), although the BALF titer for *∆potE ∆speF* was slightly lower at the 30-h timepoint. These data suggest little to no role for the *potE-speF* operon in lung infection, sharply contrasting with the OM model that, instead, showed much higher disease burden in the double knockout mutant.

### Middle ear fluids have increased alkalinity when infected with *H. influenzae*

While testing the WT and *∆potE ∆speF* strains for pathogen burden in the mouse OM model, we also measured the pH of the middle-ear washes, finding that they had significantly increased pH after infection by the *∆potE ∆speF* mutant compared to WT (Fig 7A). Although this result initially seemed inconsistent with the pH-increasing effects of *potE-speF* in ornithine-supplemented *in vitro* cultures, importantly, the *in vivo* results also showed an orders-of-magnitude difference in disease burden (Fig 6A). Thus, we reason that the increased pH of middle ear fluid could be part of a host response to infection, and the increased pathogen load in the infections by *∆potE ∆speF* could explain this finding.

**Fig 7:**
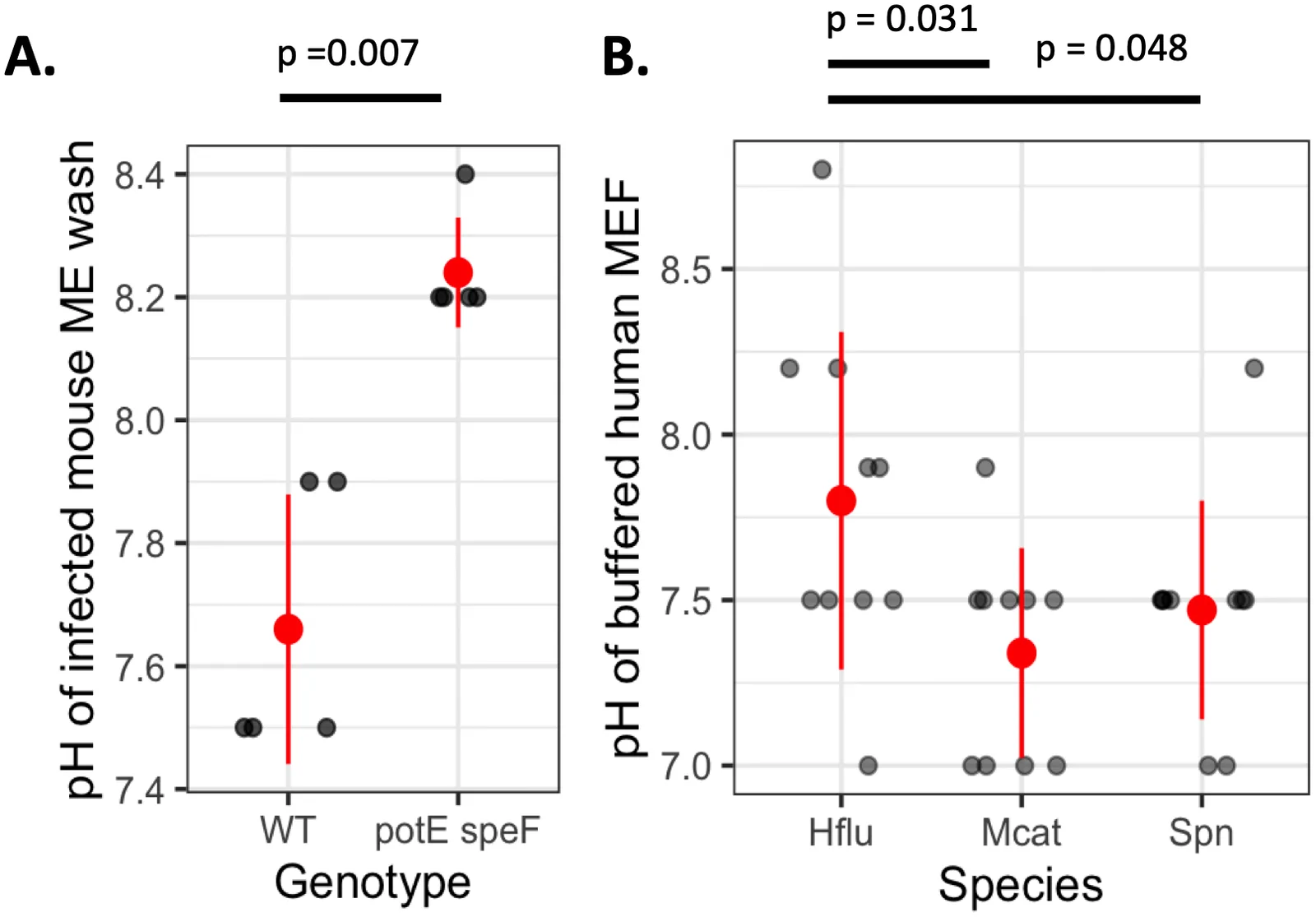
The pH of middle ear fluids during *H. influenzae* OM infection. **A.** The pH of middle ear washes from the mouse OM experiment shown in Fig 6. **B.** The pH of human middle ear fluids collected into PBS buffer that were infected by three different OM pathogens. Boxes indicate the mean, and error bars indicate the standard deviation. Statistical significance was assessed using Kruskal-Wallis test, followed by Wilcoxon Rank Sum tests to distinguish differences among groups, as indicated. Only p-values < 0.05 are shown.

Previous reports have noted that middle-ear fluids from human otitis media infections can be alkaline, raising the question of whether the pH of human MEF specimens differs depending on the infecting pathogen [54–58]. Furthermore, the association study here identified a potential role in OM disease for *H. influenzae* genes involved in pH homeostasis. To test whether the pH of infected middle ear fluid varied among the three main OM pathogens (*H. influenzae*, *S. pneumoniae* and *M. catarrhalis*), we selected 10 stored MEF specimens from the biorepository for which there was clear evidence of infection by only one of the three species. The pH of these PBS-buffered MEF specimens had marginally higher pH in the *H. influenzae* specimens compared to those of *M. catarrhalis* and *S. pneumoniae* (Fig 7B). Importantly, at collection during tympanocentesis, these specimens had been directly collected into phosphate-buffered saline (PBS) at pH 7.5 (in a ratio of ~1:10 to ~1:1, depending on the approximate volume of MEF recovered, ranging from ~50–500 µl), so the differences seen here likely underestimate the true variation in pH among specimens. Together with the high alkalinity of mouse ME washes in the high-burden infections caused by the *∆potE ∆speF* mutant, this result supports the existence of a pathogen-specific host response to *H. influenzae* infection that leads to alkaline middle-ear fluids [56], and they indicate a role for *H. influenzae* pH homeostasis genes.

## Discussion

The contribution of bacterial genetic variation to the risk of common human infections has become increasingly clear, especially since the sequencing of genomes from clinical isolate collections has become practical [23,44,90–95]. Genome sequencing of bacterial populations has not only found utility for epidemiological tracking but also for identifying potential virulence factors by association testing. Although asexual bacterial populations often have highly stratified structures, many species have sufficiently high rates of recurrent evolutionary changes to carry out association testing, due to high-frequency mutations, horizontal gene transfer, and allelic recombination mediated by natural competence and other mechanisms. Here, we used a set of carefully curated *H. influenzae* clinical isolates from persistently colonized children that had or had not experienced recurrent OM disease to carry out bacterial genome sequencing and association testing, assessing three main questions: (1) How much does genetic diversity among *H. influenzae* strains impact OM pathogenesis? (2) Can we isolate *H. influenzae* genetic variants associated with OM? and (3) what mechanisms underlie variable OM pathogenicity caused by diverse *H. influenzae*?

Association studies of *H. influenzae* accessory genes have previously compared carriage isolates from the nasopharynx to those collected from a variety of disease states [23]. Although these studies used older microarray technology and had not controlled for population structure, they implicated several accessory genes in pathogenesis. One positive association with disease was with a family of accessory genes containing Sel1-like repeats (so-called vSLRs), which was later experimentally tested for a role in pathogenesis. One of these loci, *msf*, was found to confer increased survival within macrophages, as well as higher rates of systemic infection in the chinchilla animal model of OM [44]. The molecular function of *msf* remains unknown, but these studies validate the overall approach and underline the role of bacterial variation accessory gene content in modulating a bacterium’s virulence potential.

Because the set of genomes we produced was relatively small, we focused on identifying differences in gene content associated with disease, despite likely missing important contributions from the allelic variation. Also, rather than using phenotypic traits that were directly measured from the isolates themselves, we sought associations with clinical source, which may have reduced our power to detect associations due to the multifactorial nature of infection that also depends on many host factors, while favoring identification of clinically relevant differences. Since this study focused on isolates from healthy and OM-prone children, additional work would be needed to determine whether the associations we identified here are specific to recurrent OM disease or are more generally associated with sporadic OM or chronic disease. Although some genotypes were oversampled at certain subject-timepoint combinations, the seven candidate genes/operons that we identified were supported not only by naïve statistical tests but also when accounting for gene-trait co-occurrence on a species-level phylogenetic tree. Of the newly identified associations between *H. influenzae* accessory gene content and OM disease, five of seven were between OM and the absence of a gene/operon. Remarkably, all five had predicted functions consistent with roles in nitrogen cycling, polyamine metabolism, and pH homeostasis. Changes in pH have been showed to affect *H. influenzae* behavior, especially in the context of biofilm formation [36,58,59,83], and polyamines are known to play roles in pathogenesis in other organisms [96,97].

The function of the candidate “anti-OM” operon *potE-speF* was confirmed to confer on bacteria the ability to take up ornithine and secrete putrescine (ornithine-putrescine antiporter), followed by conversion of ornithine into putrescine (*i.e.,* ornithine decarboxylase, or ODC) – thereby increasing the pH of the surrounding environment. Notably, ODC activity (in addition to indole production and urease activity) was originally part of an early ‘biotyping’ scheme used to phenotypically distinguish among *H. influenzae* clinical isolates. This predates the genetic typing methods that later became common and more accurately capture the relationships among strains [98–104]. Because many of these studies were looking for associations of disease with distinct ‘biotypes’, it remains unclear whether ODC activity by itself was associated with specific health or disease states in the older work.

A study of iron/heme-regulated gene expression in three strains previously found that two strains lacking *potE-speF* had in its place *hgpA*, which encodes a hemoglobin/haptoglobin binding protein, and furthermore both were controlled by the same iron/heme-regulated promoter [105]. This suggests the *potE-speF* operon is induced under iron/heme or oxygen limitation [106], but it also raises the possibility that the statistical association of *potE-speF* is entangled with effects of *hgpA* (or its paralogs *hgpB* and *hgpC*). However, this mutually exclusive pattern did not hold across the set of genomes in this study. All strains had multiple copies of an *hgp* homolog, and only nine had evidence for mutual exclusivity with *potE-speF*. Furthermore, inactivation of *potE-speF* from the laboratory Rd KW20 strain not only blocked alkaline-induced cell death in the presence of ornithine, it also drastically increased disease burden in a mouse OM – but not lung – infection model. This demonstrates that *potE-speF* is not a general “anti-virulence” operon but instead appears to confer fitness defects specifically in the context of OM.

We were initially surprised that the pH of mouse OM middle ear washes had higher pH when infected with the ∆*potE ∆speF* deletion mutant than with wild type, but we suggest that if increased alkalinity in middle ear fluid is primarily a host response, then the massive difference in disease burden explains the result. Since mice infected with mutant bacteria had much higher pathogen levels than those infected with wild-type bacteria, we reason that a host response that involves increasing the pH of middle ear fluid would reconcile the *in vitro* and *in vivo* observations. That is, strains carrying *potE-speF* self-limit upon encountering host-produced middle-ear fluids by exacerbating the alkaline stresses they experience, whereas strains lacking *potE-speF* can sustain growth in the infected middle ear environment, thus eliciting a stronger host response. Why middle-ear fluids sometimes become alkaline, how host cells contribute, how alkalinity affects the immune-inflammatory response, and what the underlying mechanisms are all remain unknown. Nevertheless, our finding that human middle ear fluid pH was higher on average for *H. influenzae* infections over those of the other two main OM pathogens likely supports a specific host response to *H. influenzae* infections.

The association of bacterial gene absences or loss-of-function mutations with infectious disease has been observed in other host-pathogen systems and has been referred to as “anti-virulence” or “pathoadaptation”. One classic example is the convergent loss of lysine carboxylases (LDC, encoded by *cadA*) upon independent acquisitions of shiga toxins by *Escherichia coli* to become *Shigella*. LDC converts lysine to cadaverine, which inhibits shiga toxin activity and blocks release of *Shigella* cells from host intracellular vesicles [107–109]. This provides an explanation for how the selective benefits of expressing shiga toxins are improved by loss of *cadA*. However, the *cadABC* system also provides acid tolerance to *E. coli*, other Enterobacteraceae and *Helicobacter*, since LDC activity consumes protons. Therefore, in the context of acid stress, the presence of this system instead provides a selective benefit [110].

In addition to the *potE-speF* operon*,* another especially strong association observed between OM disease and accessory gene content was to *iciA-cadA*, which is relatively rare in *H. influenzae* (and, notably, is absent from the lab strain Rd KW20 used for mutant analysis). These genes are predicted to encode a LDC (CadA), along with a transcriptional regulator predicted to control its own operon (IciA). This association parallels that of *potE-speF* with respect to a role in pH homeostasis, which may have antagonistic fitness consequences in different environments. It also further highlights a role for bacterial pH homeostasis and polyamine metabolism in OM disease caused by *H. influenzae*. However, the activities of both the CadA and SpeF proteins also impinge upon flux through central metabolic pathways, since both use as their substrates molecules that emerge from amino acid biosynthesis steps that branch from the tricarboxylic acid pathway. Notably, in *H. influenzae*, this pathway is incomplete and does not form the typical cycle, since it lacks genes encoding three enzymes (citrate synthase, aconitase, and isocitrate dehydrogenase) [84]. Interestingly, our LCMS analysis found that in the absence of *potE-speF*, cadaverine levels were nearly absent, indicating that *potE-speF* affects cadaverine production, perhaps because SpeF itself has a broad substrate specificity or via altered flux through an unknown alternative cadaverine production pathway. Thus, although both the absence of *potE-speF* and *iciA-cadA* are associated with OM and likely affect pH homeostasis, other indirect effects acting through central metabolism cannot be excluded, and the phenotypic effects of *potE-speF* and the other candidates have been only partially explored.

The single positive association of an accessory gene with OM detected in this study encodes the toxin of a recently described competence-regulated toxin-antitoxin system in *H. influenzae* [74]. That study shows this operon possesses two promoters: a constitutive promoter that is active in log-phase cells that starts within the toxin gene, and a second upstream CRP-S promoter induced under competence conditions that leads to intact toxin and antitoxin production. Antitoxin mutants with unopposed toxin have no observable growth or viability defects, but natural competence is blocked, likely due to a non-specific RNase activity of the toxin. In the same study, a survey of 181 genomes found that although most strains (89.5%) had an intact antitoxin, only 13.3% also had an intact toxin [74]. Although the function of this and other toxin-antitoxin systems remains elusive in the absence of a known secretion system to deliver the toxin to other cells, the expression of this system in nutrient-limited conditions may modulate growth or survival of bacterial cells in the infected middle ear.

Other loss-of-function changes associated with disease have also previously been seen in *H. influenzae*. In a previous study of recurrent genetic changes during persistent COPD lung infections, we identified loss-of-function mutations in the bifunctional *ompP1/fadL* gene that arise in the course of persistent infections. In that case, loss-of-function mutations were from short frameshift and/or premature stops, which likely had recently arisen in the course of long-term persistent infection of the lungs due to natural selection for resistance to the inflammatory mediator arachidonic acid [45]. In contrast to the long-term evolution of *H. influenzae* during COPD, we think that the genes identified in this study whose absences are associated with OM disease did not arise within individual pediatric subjects. Instead, we speculate that strains that carry the *potE-speF* genes and other “anti-OM” candidates have low fitness in the middle-ear environment due to maladapted pH homeostasis under alkaline stress, thus they are rapidly cleared by natural selection. Further investigation of this and other natural bacterial variants associated with OM disease will give insights into molecular mechanisms of pathogenesis, and they may provide for new diagnostics to evaluate pathogenesis risk or new treatment approaches to preventing recurrent and chronic disease.

## Materials and methods

### Ethics statement

The long-term sampling study was approved by the Institutional Review Board of the Rochester General Hospital Research Institute (approval number 1141B), and written informed consent was obtained from parents prior to child enrollment.

### Bacterial cultures

*H. influenzae* were cultured using standard methods [111,112]. Liquid cultures were grown at 37ºC and 5% CO_2_ on a roller drum or shaking at 200 RPM in sBHI, brain-heart infusion broth (Difco) supplemented with hemin (10 μg / mL; ICN Biochemicals) and β-nicotinamide adenine dinucleotide (NAD) (2 μg / mL; Sigma). Solid media cultures were grown in a 37°C incubator with 5% CO_2_ on chocolate (Biomérieux) or sBHI (Condalab) agar. Erythromycin (11 µg / mL; Erm_11_) was used to supplement media for *H. influenzae* as required. *Escherichia coli* was grown in Luria Bertani (LB) broth or on LB agar at 28°C or 37°C, supplemented with ampicillin (100 µg / mL; Amp_100_) or erythromycin (150 µg / mL; Erm_150_) as required.

### Study population and clinical isolates of *H. influenzae*

Clinical isolates of *H. influenzae* were retrospectively selected from the biorepository at the Rochester General Hospital Research Institute. These had originally been collected from pediatric subjects between ~6–36 months of age between mid-2006 and late 2016, as previously described [61,62,113]. Enrolled subjects were prospectively sampled at six regularly scheduled pediatric checkups (“healthy” visits), as well as at any acute OM episodes (“AOM” visits) they experienced. At any visit, nasal wash specimens were collected, along with nasopharyngeal and throat swabs. At AOM visits, middle ear fluid specimens were also collected into phosphate-buffered saline (1xPBS) from one or both ears by tympanocentesis, as indicated, and standard-of-care treatment was prescribed. Approximately a week after AOM episodes, follow-up visits were also conducted with sampling as above. Follow-ups were typically labeled as “healthy” visits unless also associated with a persistent or rapidly recurring infection. Nasal wash and middle ear fluid (MEF) samples were subsequently partitioned into three aliquots: one directly plated for bacterial isolates, a second inoculated into overnight broth cultures that were stored and then plated for bacterial isolates, and a third into Trizol for later bulk analysis of the original specimen. For conciseness, we collectively refer to isolates originally collected from the throat, nasopharynx, nasal washes, or nasal wash broths (*i.e.,* not from the middle ear) as “NP” isolates, while those collected from middle ear fluids, either directly or after overnight culture, as “MEF” isolates. Individual isolates were cultivated, identified, and stored. Typically, only a single isolate was picked for a given species from a given specimen, but several isolates of the same species were often collected from distinct specimens taken from the same subject and time point. In addition to *H. influenzae*, *S. pneumoniae*, *M. catarrhalis*, and alpha-hemolytic *Staphylococcus* were also commonly isolated.

At the time clinical isolates were retrospectively sampled (late 2016), the collection comprised bacterial isolates from 1178 subjects spanning 5545 doctor visits and 1410 OM episodes, with 2271 *H. influenzae* strains isolated from 1083 doctor visits, which included 579 OM episodes. Longitudinal patient series were selected from the total collection based on the following criteria: (a) approximately half from OM-prone children (OP, or stringently-defined recurrent acute OM, defined as experiencing ≥3 episodes within 6 months or ≥4 within 12 months along with microbiological confirmation from MEF [13,63,113]) and half from healthy children (NOP, at most 1 OM episode with no *H. influenzae* isolation); and (b) high prevalence of *H. influenzae* across the series (≥3 healthy visits with an *H. influenzae* isolate and, for OP subjects, ≥ 2 healthy visits and ≥3 OM episodes with *H. influenzae* isolation). This resulted in a set of 109 longitudinal series, from which 21 were randomly chosen (9 from NOP and 12 from OP subjects). This set had originally comprised 267 specimens collected from the 21 subjects at 123 distinct visits. Details about subjects and individual isolates that were successfully sequenced are in **Supplementary** S1 and S2 Tables.

### Whole-genome sequencing and clonal lineage assignments

Of the original 267 specimens, 223 single-colony isolates were successfully revived from cryo-storage, cultured, and had high-quality genomic DNA extracted. *S*tocks were streaked on chocolate agar petri dishes and grown overnight in a 37°C incubator with 5% CO_2_. For most isolates (n = 204), a single colony was picked and grown overnight in 5 mL sBHI in a 37°C shaker (250 rpm). Two 1 ml aliquots were used for 15% glycerol stocks at -80°C, and the remaining cells were pelleted at 3000 RPM for 5 min, decanted, and stored at -80°C for DNA extractions. For isolates with poor growth (n = 19), cells were scraped off lawns grown on chocolate agar, resuspended in sterile water, pelleted at 3000 RPM for 5 min, and stored at -80°C for DNA extraction. Genomic DNA was extracted from pellets using DNeasy Blood & Tissue Kits (QIAGEN). DNA integrity was verified by agarose gel electrophoresis, purity was evaluated spectrophotometrically (NanoDrop 1000, Thermo Scientific), and concentration was estimated fluorometrically (Synergy HT plate reader, BioTek; Quant-It dsDNA Assay Kit, Molecular Probes).

DNA extractions were processed into Illumina sequencing libraries using Nextera XT tagmentation according to manufacturer guidelines (1 ng genomic DNA input), followed by pooling libraries to 96-plex, and sequencing pools on a NextSeq 500 using mid-output runs at 2x150 nt, targeting an average coverage of ~200-fold per genome. The genome assembly pipeline was as previously described [45]; briefly, technical sequence removal used Trimmomatic (v 0.33) [114], overlapping paired-ends were merged using COPE (v 1.1.2) [115], error-correction used a module from Allpaths-LG [116], and sequence assembly used SPADES (v 3.7.0) [117]. Contigs were removed if their length was ≤ 256 bp, > 98% of bp were homopolymers, or coverage was < 10-fold per million reads. Further quality control removed assemblies with >100 contigs, assembly lengths of <1.7 Mb or >2.3 Mb, and assemblies that were not identified to the species level with high confidence as *Haemophilus influenzae* by Taxator-tk (v1.2) [118]. In addition, polyclonal and contaminated samples were identified using ConStrains (v. 0.1.0) [119]. After excluding poor and contaminated assemblies, we used a set of 206 genomes from 120 visits for further analysis.

After clonal lineage (CL) assignments using the Illumina assemblies (see below), a subset of 39 genomic DNAs representing each CL with >1 isolate in the collection were also sequenced using long-read Pacific Biosciences (PacBio) technology, either RSII or Sequel. SMRTbell libraries were prepared according to PacBio recommendations and pooled to target >50-fold genomic coverage. RSII data were assembled using HGAP2, and Sequel reads were assembled using Falcon. Polishing used Circlator (v1.0.2) [120] (with permutation to the *dnaA* gene) with error correction at the permuted junction using the PacBio resequencing pipeline. For PacBio assemblies that did not circularize, assemblies were still permuted to *dnaA* by splitting single un-circularized contigs into two at *dnaA* and arranging these to the ends of the assemblies. Assemblies used the similar quality control criteria as for Illumina-based assemblies, except required <10 contigs after removing those with low quality scores (<35 quality for RSII; < 40 quality and <0.15 coverage for Sequel). Final error correction used Pilon with Illumina reads against the PacBio assemblies.

Clonal lineages (CLs, sets of closely related strains) were determined using three related methods. PopPunk was used to define clusters of strains based on a combination of nucleotide divergence and genomic content differences (minimum k-mer = 15 under the DBSCAN clustering model without refinement) [67]. Mashtree was also applied, also including an additional 38 complete *H. influenzae* genomes from RefSeq [121]. Multi-locus sequence type (MLST) was determined from the assemblies using Torsten Seeman’s tool https://github.com/tseemann/mlst (v. 2.11) [122] to access the pubMLST.org database [123]. All three lineage assignment methods largely agreed, although MLST assignments failed to classify some strains to an existing MLST due to novel alleles of some MLST genes not in the database.

### Genome annotation, gene clustering, and phylogenetic inference

Genome annotation, pan-genome clustering, and phylogeny inference followed previous methods [45]. In brief, gene annotations used Prokka (v1.11) [124], and clusters of homologous protein-coding genes were identified using Roary (v3.5.1) [64] with a blastp threshold of 75%. Clusters with at most one gene per strain were then re-aligned using the codon-aware aligner Prank [65], and alignments were concatenated for phylogeny inference with RAxML using the GTRCAT model (v8.2.4) [66,125]. RAxML treats gaps (*i.e.,* indels and single-copy genes missing from one or more strains) as missing data. The tree was midpoint rooted, since this agreed with the root of outgroup-rooted trees that included sister species *H. haemolyticus* and *H. parainfluenzae* but included only ~10% of the number of genes.

### Accessory gene association tests

To identify associations between gene possession (presence or absence of a protein-coding gene) and clinical isolation from health or disease, clinical isolates were classified by three clinical provenance “traits”: (1) isolation site, MEF *vs.* NP; (2) visit type, acute OM *vs.* healthy); and (3) subject class (OM-prone, *vs*. not OM-prone). Treatment outcome (success *vs.* failure) included only a few isolates, so these were not subjected to further analysis. For each of the three clinical provenance traits, we used Scoary [71] with our phylogenetic tree inference and the Roary gene presence/absence matrix, allowing for identically distributed accessory genes to be collapsed using the --collapse option. Results were deemed significant if the Benjamini-Hochberg adjusted p-value was < 0.05 and for which gene-trait co-occurrences on the phylogenetic tree gave a “best”-case binomial test p-value < 0.05.

### *H. influenzae* gene inactivation

Strains, plasmids, and primers used are in **Supplementary** S5 Table. We generated ∆*potE* and *∆potE-∆speF* knockouts in the laboratory strain Rd KW20. For disruption of *potE* (locus ID HI0590), a DNA fragment containing *potE* and its flanks (2,380 bp) was PCR amplified with Phusion polymerase (ThermoFisher Scientific) using Rd KW20 genomic DNA as a template and primers *potE*-F1 and *potE*-R1. This fragment was cloned into pJET1.2/blunt, generating pJET1.2-*potE*, followed by disruption of the cloned product by inverse PCR using primers *potE*-F2 and *potE*-R2. An internal 43-bp fragment was replaced by a blunt-ended Erm^R^ cassette excised by *Sma*I digestion from pBSLerm [126], generating pJET1.2-*potE*::*ermC*. This plasmid was used as a template to amplify the *potE::ermC* disruption cassette with primers *potE*-F1 and *potE*-R1. For disruption of the *potE-speF* locus (HI0591 and HI0590), a DNA fragment containing both genes and flanking sequences (3,575 bp) was PCR amplified using Rd KW20 gDNA as template and primers *speFpotE-*F1 and *speFpotE*-R1. The fragment was cloned into pJET1.2/blunt, generating pJET1.2-*speF-potE*, and the cloned product was disrupted by inverse PCR using primers *speFpotE*-F2 and *speFpotE*-R2. An internal 351-bp fragment was replaced by a blunt-ended Erm resistance cassette excised by *Sma*I digestion from pBSLerm, generating pJET1.2-*speFpotE*::*ermC*. This plasmid was used as a template to amplify the *speFpotE::ermC* disruption cassette with primers *speFpotE*-F1 and *speFpotE*-R1. Both *potE* and *speFpotE* disruption cassettes were used to transform the RdKW20 strain using the MIV method to induce natural competence. Transformants were selected on sBHI agar + Erm_11_ to select for Rd KW20 Δ*potE* and Rd KW20 Δ*potE* Δ*speF* mutants [111]. In all cases, mutants were confirmed by PCR. We also attempted to knock out the *speF* gene alone in Rd KW20 by a comparable approach but no transformants were recovered that had correctly targeted resistance cassettes, suggesting that knockout of Δ*speF* in a strain expressing *potE* are inviable.

### Genome sequencing analysis of the Rd KW20 WT and Δ*potE* Δ*speF* MUT strains

Single-colony passages of the WT and MUT strains were grown in 5-ml overnight cultures in sBHI, and genomic DNA was subjected to PacBio Sequel IIe sequencing after SMRTbell library prep v2.0 with a target coverage of ~350-fold genomic coverage by HiFi reads. The original Rd KW20 genome assembly (GenBank accession L42023.1) was modified to convert non-ACGTN characters into Ns and permute the genome to the *dnaA* gene. HiFi reads were then aligned to this reference using minimap2 [127] under recommended settings for HiFi reads. Reads spanning the breakpoint junctions at *potE-speF* were used to confirm the fixed insertional replacement in the mutant *speFpotE*::*ermC* by manual inspection of alignments with IGV. Additional differences in the distribution of variant alleles between strains were identified using FreeBayes with PacBio HiFi settings and in ‘pooled-continuous’ mode [128]. Variants showing strongly skewed allele frequencies between strains were further investigated, and the impact of each variant was determined by manual inspection against the Rd reference annotation.

### Growth curve assays in varying pH and polyamine supplementation

To monitor the effects of pH and polyamine supplementation on bacterial growth, cultures were inoculated 1:50 into sBHI with cells revived from frozen log-phase stocks (frozen in 15% glycerol stocks at OD_600_ = 0.3, defrosted, and resuspended in fresh sBHI) and grown in multi-well plates (Corning, Inc, Durham, NC) in a plate reader (Synergy HT, BioTek) at 37°C with medium shaking. Growth over time was determined by readings of optical density at 600 nm (OD_600_) taken by the plate reader every 15 min for 18 h. Prior to inoculation, sBHI culture media was supplemented with varying concentrations of polyamines (ornithine, putrescine, L-ornithine and putrescine at 1:1, cadaverine, spermine, spermidine, and tyramine at 0, 12.5, 25, or 50 mM), followed by pH adjustment to 6, 7.5, or 9 using 3N hydrogen chloride or sodium hydroxide. Determination of pH before and after growth used pH paper (pHydrion papers; Micro Essential Laboratory Inc, Brooklyn, NY).

### Growth and survival in varying pHs with ornithine supplementation

Cultures were inoculated with log-phase stocks at 1:25 into 5 mL pH-adjusted sBHI cultures ± 50 mM L-ornithine and incubated in a 37°C shaker (250 RPM). At 4, 5, or 18 h, aliquots were removed for: (a) measurement of pH, (b) bacterial titers of colony-forming units per ml (CFU/mL), and (c) extraction of polyamines for liquid chromatography-mass spectrometry (LC-MS). CFU/ml titers were determined by plating 50 μL of 4 cell dilutions (10^-5^, 10^-6^, 10^-7^, 10^-8^) on sBHI plates and counting colonies after overnight growth in a 37°C incubator with 5% CO_2_. Individual replicates (n = 4) were performed on separate days. Significant differences were determined by multi-factor ANOVA of log-transformed CFU/ml given genotype (WT Rd KW20 Δ*potE*, and Δ*potE* Δ*speF*), supplementation (± 50 mM ornithine), and starting pH (6, 7.5, or 9); differences were considered significant for p < 0.05.

### Mass spectrometric analysis of polyamine levels in bacterial cell pellets and spent media supernatants

For extractions of polyamines for LC-MS analysis [129–131], 1 mL of culture was pelleted, supernatants were set aside, pellets were washed once in PBS, and both pellets and supernatants were stored at −20°C. After samples were thawed, supernatants were lyophilized, and both pellets and lyophilized supernatants were resuspended in 200 μL MOPS buffer, followed by 3 cycles of freeze-thaw using liquid nitrogen alternating with a 75°C water bath. Trichloroacetic acid (60 µl) was added, and tubes were incubated on ice for 5 min before centrifugation at 13,000 RPM for 5 min to pellet debris. Final supernatants were collected and stored at -20°C until preparation for LCMS.

For LCMS, polyamine standards and extracts of pellet and supernatant were pre-column-derivatized by addition of 5% v/v benzoyl chloride in acetonitrile and 100 mM sodium carbonate. Standards included ornithine, putrescine, cadaverine, spermine, spermidine, diaminopropanol, and tyramine, as previously detected in Pasteurellaceae species [87]. Pre-column-derivatized polyamine standards and pellet/supernatant extracts were analyzed on a Waters Acquity I-Class UPLC system coupled to an Acquity TUV detector and Synapt G2Si HDMS mass spectrometer in positive ion mode with a heated electrospray ionization source in a Z-spray configuration. LC separation was performed on a Waters Acquity UPLC BEH 1.7-µm, 2.1-mm, x 50-mm column using an 0.6 mL / min gradient of 95/5–15/85 A/B in 4 min followed by washing and reconditioning the column. Eluent A was 0.1% formic acid in water and B was 0.1% formic acid in acetonitrile. Conditions on the mass spectrometer were as follows: capillary voltage 0.5 kV, sampling cone 40 V, source offset 80 V, source 120°C, desolvation 250°C, cone gas 0 L / h, desolvation gas 1000 L / h, and nebulizer 6.5 bar. The analyzer was operated in resolution mode and low-energy data were collected between 100 and 2000 Da at 0.2-sec scan time. MS^e^ data were collected using a ramp trap collision energy of 20–40 V, and masses were extracted from the time-of-flight MS total ion currents using an absolute width of 0.005 Da.

Peak areas corresponding to each polyamine on the panel served as relative measures of polyamine concentration in the cells (pellets) and extracellular environment (lyophilized media supernatants). Significant differences were determined for each polyamine at each time point by multi-factor ANOVA of each polyamine’s peak area given the genotype (WT or Δ*potE* Δ*speF*), fraction (pellet or supernatant), supplementation (± 50 mM ornithine), and starting pH (6, 7.5, or 9); differences were considered significant for p < 0.05.

### Mouse model of acute otitis media infection by *H. influenzae*

To assess the disease burden of *H. influenzae* in an animal model of acute OM, we employed an established influenza virus co-infection model [88]. C75Bl/6 mice were housed under pathogen-free conditions at the animal facilities at RGHRI and used at 6–8 weeks of age. Animal handling and procedures were in accordance with the Institutional Animal Care & Use Committee at RGHRI. Bacterial strains were grown in sBHI in a 37°C shaker (250 RPM) to an OD_600_ of 0.6 and frozen at -80°C with 15% glycerol until use. Bacterial CFU/ml titers were determined when stocks were thawed. On Day 0 after acclimatization, mice (n = 4) were inoculated intranasally with influenza virus PR8 (TCID_50_ = 10 µL). On Day 7, *H. influenzae* was intranasally inoculated into anesthetized mice at 5 x 10^7^ CFU / dose. On Day 10, mice were sacrificed and *H. influenzae* burden was measured in nasal lavages, ME washes, and combined ME bullae homogenates. Infections were performed in groups of 4 mice per genotype. Significant differences between genotypes collectively or for each specimen type alone were evaluated by one-way ANOVA on log-transformed CFU measurements, followed by Tukey’s HSD (“honest significant differences”) as indicated, and differences were considered significant for p < 0.05. The pH of ME washes was measured using pH test strips (7.5 to 9.5 range and 0–14 range; VWR Chemicals BDH), and significant differences (p < 0.05) were evaluated using Kruskal-Wallis tests and pairwise Wilcoxon rank sum tests.

### Mouse model of lung infection by *H. influenzae*

For mouse lung infections, animal handling and procedures were in accordance with European (Directive 2010/63/EU) and National (RD118/2021) legislation, with authorization of the CSIC Animal Experimentation Committees and local Government (Protocol PI007/19). To determine differences in infectious burden during lung infection, a mouse model of *H. influenzae* lung infection was used, as previously described [132–136]. CD1 female mice were purchased from Charles River Laboratories (France), housed under pathogen-free conditions at the Institute of Agrobiotechnology facilities (registration number ES/31-2016-000002-CR-SU-US), and used at 22–25 g (6–7 weeks). Bacterial strains were revived and grown on chocolate agar before scraping from the plate and making a bacterial suspension in sBHI. For intranasal infection, 20 µL of a suspension containing 5 x 10^9^ CFU/mL (1 x 10^8^ CFU / mouse) was placed at the entrance of the nostrils until complete inhalation by the mouse, while the mouse was anesthetized (ketamine-xylazine, 3:1). At 24 or 30 h post-infection (hpi), mice were euthanized using cervical dislocation. BALF samples were obtained by perfusion and collection of 0.7 mL of PBS, with the assistance of a sterile 20G (1.1-mm-diameter) Vialon intravenous catheter (Becton-Dickinson) inserted into the trachea. Recovered BALF was serially 10-fold diluted in PBS and plated in triplicate on sBHI agar to determine the number of viable bacteria. By following published procedures [137], we determined a limit-of-detection of < 3–4 CFU/mL BALF. The left lung was individually weighed in sterile bags (Stomacher 80, Seward Medical) and homogenized 1:10 (w/v) in PBS. Each homogenate was serially 10-fold diluted in PBS and plated in triplicate on sBHI agar to determine the number of CFU per lung. Infections were performed in groups of ≥ 5 mice per genotype and time point. Significant differences among genotype, specimen type, and time point (BALF and lung homogenates) were evaluated by multi-factor ANOVA on log-transformed CFU/ml measurements, and differences were considered significant for p < 0.05.

## Supporting information

S1 Table Subject Information. OP for otitis prone, NOP for not otitis prone. All subjects were sampled between 6 months and 3 years of age.
(XLSX)

S2 Table Collection and genome metrics for clinical isolates of *Haemophilus influenzae.* Isolate_ID through B_Number indicate different strain tracking IDs. Cohort: OP for otitis prone; NOP for not otitis prone. Visit type: Well Visit was during a regular pediatric check-up with no OM; sick visits were during OM episodes; Follow up was around a week afer an OM episode. Collection_Site: NP means the isolate was collected from the upper airway not the middle ear; ME means the isolate was collected from middle ear fluid taken by tympanocentesis. Sample_Type: NW(B) for nasal wash (broth); NP for nasopharyngeal swab; TC for throat culture; LMEF(B) for left middle ear fluid (broth); RMEF(B) for right middle ear fluid (broth). Other_Species_Isolated indicates concurrent isolation of other microbes from the same specimen: None for only *H. influenzae* isolated; Spn for *Streptococcus pneumoniae*; Mcat for *Moraxella catarrhalis*; Hpi for *H. parainfluenzae*; Ahs for alpha-hemolytic *Staphylococcus*; Other for none of the above or not identified.
(XLSX)

S3 Table Scoary results. Scoary was run using Roary outputs for each of three comparisons depending on clinical isolation source: NP vs. ME, healthy vs. sick subject, OP vs. NOP subject (only OP subjects had *H. influenzae* isolates from ME or sick but both had healthy and NP isolates). Results were filtered to those with Fisher’s exact test FDR adjusted p-value < 0.05 and Best_pairwise_comp_p < 0.05. “Scoary-collapsed” refers to testing genes with identical distributions across strains together, separated by triple hyphens to clarify co-inheritance and minimize unnecessary tests. Odds ratios are reported with the first listed isolation source as the numerator.
(XLSX)

S4 Table Five second-site variants with asymmetric allele distributions between WT (Rd KW20) and MUT (*∆potE ∆speF*). Each strain was single-colony passaged, and DNA was extracted for PacBio HiFi sequencing to ~300–400-fold genomic coverage. After minimap2 read alignment to the Rd KW20 reference sequence and FreeBayes variant calling in ‘pooled-continuous’ mode, 22,823 positions were identified with at least one read in one sample supporting an alternate (ALT) allele. Of these >99.9% showed agreement in reference (REF) allele frequencies between the WT and MUT, most agreeing with the REF allele at >75%. A small set (n = 536 positions) supported an ALT allele in both WT and MUT, consistent with previously documented reference errors. An additional site was at ~50% allele frequency in both strains. The remaining 5 variants with asymmetric REF allele frequencies are shown in the table. POS is against an Rd KW20 reference permuted to the *dnaA* gene. GT refers to the FreeBayes-determined genotype, *i.e.,* 0 = REF, 1 = ALT1, *etc.* WT freq and MUT freq indicate the allele frequency of each allele.
(XLSX)

S5 Table Strains, plasmids, and oligonucleotides used. (XLSX)

S1 Fig Phylogeny of Complete Genomes from Rochester repository and NCBI. A mashtree of PacBio-sequenced isolates from the Rochester collection (those with “mpi” labels), along with other complete genomes from NCBI. This tree illustrates that strains collected from children in Rochester are phylogenetically interspersed with strains with distinct geographic and clinical provenance.
(TIFF)

S2 Fig Gene synteny between the *arcB* and *cpdB* genes. The local gene neighborhood around *potE-speF* was extracted from the 44 genomes that contained the genes, and the nearest single-copy core genes that consistently flanked them were *arcB* (ornithine carbamoyltransferase) and *cpdB* (2,’3’-cyclic-nucleotide 2’-phosphodiesterase). Of 206 assemblies, *arcB* and *cpdB* were found on the same contig and with 40-kb on 126. These intervals are shown in the Fig with core and near-core genes with clear names labeled. No other partial or fragmented *potE* or *speF* genes were found with tblastn on any assemblies; the only other alignments were to the distantly related *cadA* and *cadB* genes with only ~35% amino acid identity.
(TIFF)

S3 Fig Growth curves in the presence of increasing concentrations of polyamines in two genotypes at 3 pHs. Putrescine-L-ornithine was with both polyamines at 1:1 totaling the indicated concentration.
(TIFF)

S4 Fig Growth curves in the presence of increasing concentrations of polyamines in two genotypes at 3 pHs. DAP is diaminopropanol.
(TIFF)

S5 Fig Change in pH after 18 h culture with varying concentrations of six polyamines. A. pH change for growth curves shown in Supplementary Fig S2. B. pH change for growth curves shown in Supplementary Fig S3.
(TIFF)

S6 Fig Ornithine levels in two genotypes, 3 pHs, and with/without ornithine supplementation at 2, 4, 5, or 24 h. (TIFF)

S7 Fig Putrescine levels in two genotypes, 3 pHs, and with/without ornithine supplementation at 2, 4, 5, or 24 h. (TIFF)

S8 Fig Cadaverine levels in two genotypes, 3 pHs, and with/without ornithine supplementation at 2, 4, 5, or 24 h. (TIFF)

S9 Fig Spermidine levels in two genotypes, 3 pHs, and with/without ornithine supplementation at 2, 4, 5, or 24 h. (TIFF)

S10 Fig Spermine levels in two genotypes, 3 pHs, and with/without ornithine supplementation at 2, 4, 5, or 24 h. (TIFF)

S11 Fig Diaminopropane levels in two genotypes, 3 pHs, and with/without ornithine supplementation at 2, 4, 5, or 24 h. (TIFF)

S12 Fig Tyrosine levels in two genotypes, 3 pHs, and with/without ornithine supplementation at 2, 4, 5, or 24 h. (TIFF)

S1 File Newick string of the phylogenetic tree built with RAxML from all single-copy genes for 206 H. influenzae isolates, including bootstraps. (NWK)
